# Prognostic Determinants of Anterior Large Vessel Occlusion in Acute Stroke in Elderly Patients

**DOI:** 10.3390/geriatrics9010013

**Published:** 2024-01-15

**Authors:** Takashi Mitsuhashi, Kohsuke Teranishi, Joji Tokugawa, Takumi Mitsuhashi, Makoto Hishii, Hidenori Oishi

**Affiliations:** 1 Department of Neurosurgery, Juntendo University Nerima Hospital, Tokyo 177-8521, Japan; j.tokugawa@juntendo-nerima.jp (J.T.);; 2 Department of Neurosurgery and Neuroendovascular Therapy, Juntendo University School of Medicine, Tokyo 113-8421, Japan; kterani@juntendo.ac.jp (K.T.);

**Keywords:** mechanical thrombectomy, large vessel occlusion, elderly, prognosis

## Abstract

This study investigated prognostic factors in elderly patients (80 years and older) undergoing mechanical thrombectomy (MT) for anterior circulation large vessel occlusion (LVO) in acute stroke treatment. Of 59 cases, 47.5% achieved a favorable outcome (mRS ≤ 3) at three months, with a mortality rate of 20.3%. Factors associated with better outcomes included younger age, lower admission National Institute of Health Stroke Scale (NIHSS) scores, lower N-terminal pro-brain natriuretic peptide (NT-proBNP) and D-dimer levels, the presence of the first pass effect (FPE), and successful recanalization. However, logistic regression showed that only lower admission NIHSS scores were significantly correlated with favorable outcomes. In addition, this study suggests that lower admission NT-proBNP and D-dimer levels could potentially serve as prognostic indicators for elderly LVO patients undergoing MT.

## 1. Introduction

Several randomized controlled trials of mechanical thrombectomy (MT) for the treatment of acute anterior circulation large vessel occlusion (LVO) have demonstrated efficacy and safety in all age groups [1]. MT has become one of the standard treatments for acute ischemic stroke caused by LVO, along with intravenous thrombolysis. Some guidelines recommend considering MT for occlusion of the M1 segment of the middle cerebral artery or internal carotid artery [2,3]. As Hendrix et al. also pointed out, although the guidelines provide a class 1 recommendation for MT in patients over 80 years of age, several RCTs have excluded patients over 80 or 85 years of age [4]. On the other hand, it is recognized that life expectancy has increased worldwide in recent years and that the risk of developing ischemic stroke is thought to increase as the number of older people increases and as they get older. Some previous studies have shown that people over 80 years of age have the highest incidence of acute ischemic stroke [5].

However, the evidence for MT in elderly patients with LVO is scarce and not explicitly stated in the guidelines. Furthermore, Alawieh et al. point out that many of the previous conclusions regarding the efficacy and safety of MT in the elderly were based on single-arm, single-institute studies, resulting in significant heterogeneity among studies and a lack of comparison with optimal medical management [6]. Previous studies on MT for LVO have highlighted advanced age as a strong predictor of unfavorable outcomes after MT [7,8].

Japan has the highest aging rate in the world, and other developed countries are also expected to become aging societies. The safety and efficacy of MT for LVO have been reported in octogenarians, nonagenarians, and elderly patients with LVO, including prognosis-related factors. Factors contributing to outcomes include successful reperfusion, the first pass effect (FPE), a low National Institute of Health Stroke Scale (NIHSS) score on admission, the initial Alberta Stroke Program Early CT Score (ASPECTS), time from onset to groin puncture, time from groin puncture to recanalization, pre-existing functional deficits, and age over 80 years [6,9,10,11,12,13]. Although the HERMES meta-analysis showed that MT had beneficial effects in patients aged 80 years or older, the proportion of cases over 80 years of age was low [1]. Therefore, the current guidelines for the United States provide a class 1 recommendation for MT in patients aged 80 years or older [2]. Accordingly, Zaidat et al. dichotomized patient age based on previous studies, using 80 years as the cut-off point and defining elderly patients as those aged 80 years or older. In the TRACK registry, age ≥80 years was reported to be a prognostic factor, and the rate of patients with an mRS score of 0 to 2 at 90 days decreased slightly with age [14]. In addition, Meyer et al. pointed out that no substantial therapy recommendation for patients aged 90+ was derived from the recent landmark thrombectomy trials [15].

In the present study, we defined “elderly” as patients over 80 years of age because, as mentioned above, many studies, including previous guidelines, often set the age at which a person is considered elderly at 80 years. Furthermore, since the average life expectancy of Japanese men is in the early 80s and that of women is in the mid-80s, we defined those aged 80 and over as elderly.

Since many elderly patients have undergone MT for LVO in our medium-sized hospital in Japan, which has an aging population, we investigated the results of MT for LVO in elderly patients and reviewed the prognostic factors to identify factors associated with favorable outcomes in patients who have undergone MT for LVO.

## 2. Materials and Methods

### 2.1. Patient Selection

A total of 141 consecutive patients who underwent MT for anterior circulation LVO at our hospital between October 2015 and October 2021 were retrospectively reviewed. The protocol of this retrospective single-center study was approved by the ethics committee of Juntendo University Nerima Hospital.

The inclusion criteria of the present study were as follows: (1) acute ischemic stroke due to LVO within the anterior circulation, including the internal carotid artery (ICA) or middle cerebral artery (MCA), with or without additional intravenous thrombolysis; (2) age at onset of 80 years or older; (3) known admission NIHSS, ASPECTS, and Diffusion-Weighted Imaging ASPECTS (DWI-ASPECTS) for patients who were eligible for magnetic resonance imaging; (4) known values of the pre-morbid modified Rankin scale (mRS) and mRS 90 days after stroke.

Data were collected based on age on admission, sex, time of onset or last known well time, time of arrival at our institute, NIHSS on admission, blood collection data on admission (N-terminal pro-brain natriuretic peptide (NT-proBNP) (pg/mL), D-dimer (µg/mL), glucose (mg/dL), and glycated hemoglobin (HbA1c) (%)), time of recanalization, number of attempts, and angiographic recanalization status as modified thrombolysis in cerebral infarction (mTICI). Stress hyperglycemia was estimated using the GAR index (glucose-to-glycated hemoglobin ratio), which was calculated using the following formula: fasting plasma glucose (mg/dL)/HbA1c (%).

In this study, we defined a clinical outcome of mRS 0–3 as a favorable clinical outcome for the following reasons.

The mRS is a non-linear scale and a measure of activity of daily life that focuses primarily on mobility, and dichotomization of outcomes has been used in research studies. Many previous studies, including HERMES, have treated mRS 0–2 as a good clinical outcome [1]. However, Wilson et al. pointed out the lower inter-rater reliability in classifying patients as mRS 2 and 3, as well as the existence of subgroups within the mRS 3 group [16]. In addition, Rangraju et al. reported that patients who achieved mRS 3 were more similar to mRS 2 than mRS 4 patients in terms of functional disability and quality of life [17].

The majority of the patients underwent MT under local anesthesia with the use of a stent retriever and/or an aspiration catheter. For the first 24 h after the intervention, all patients continued treatment in the intensive care unit. No patients required mechanical ventilation after the intervention.

### 2.2. Study Endpoints

The primary neurological endpoint of this study was the rate of favorable clinical outcomes defined as mRS ≤ 3 at the 90-day follow-up, adjusted for patient age.

### 2.3. Statistical Analysis

First, we performed a Shapiro–Wilk test for normality between the two groups with mRS 0–3: good clinical outcome and 4–6: unfavorable clinical outcome to confirm *p* < 0.05 (not normally distributed). Comparisons between variables were then performed using either the *t*-test or the Mann–Whitney U test (M-W test), as appropriate. Pearson’s chi-squared test was used for statistical validation between the two groups by gender. A binomial logistic regression model predicting favorable or unfavorable clinical outcomes was also developed and analyzed; a *p*-value < 0.05 was considered statistically significant. IBM SPSS version 25 (Chicago, IL, USA) was used for data analysis.

## 3. Results

### 3.1. Patient Characteristics

Fifty-nine elderly patients aged over 80 years met the inclusion criteria and were treated between October 2015 and October 2021.

The median age was 85 years (IQR 83–87), and 61.02% (27/59) were female. The median pre-morbid mRS was 0 (IQR 0–1), but the mean value was 0.559.

Patients were admitted to the hospital with a median NIHSS score of 24 (IQR 18–27), a median baseline ASPECTS of 9 (IQR 7–9), and a median baseline DWI-ASPECTS of 7 (IQR 6–8). Six patients were unable to undergo MRI because of metallic implants in their bodies.

The most common cardiovascular risk factors (Table 1) were hypertension (83.1%, 49/59), diabetes mellitus (20%, 12/59), hyperlipidemia (13.6%, 8/59), and atrial fibrillation (56%, 33/59). There was no statistically significant difference in cardiovascular risk factors between the favorable and unfavorable clinical outcome groups according to the *t*-test and M–W test (*p* = 0.7802 and *p* = 0.7728).

The most common site of occlusion (Table 2) was the middle cerebral artery (MCA), particularly the M1 segment (45.8%,27/59); other sites in the anterior circulation included the internal carotid artery (ICA) (22%, 13/59), the MCA M2/M3 segment (18.6%, 11/59), the cervical segment of the ICA (6.8%, 4/59), and the intracranial segment of the ICA (5%, 3/59). There was no statistically significant difference in the site of occlusion between the favorable and unfavorable clinical outcome groups according to the *t*-test and M–W test (*p* = 0.8129 and *p* = 0.7481). More than half of the patients (61.02%,36/59) underwent intravenous thrombolysis prior to MT.

The baseline characteristics on admission were compared between the favorable clinical outcome (mRS ≤ 3) and unfavorable clinical outcome (mRS > 3) groups 90 days after admission (Table 3).

In terms of age, the median age in the favorable clinical outcome group was 84 years, while the median age in the unfavorable clinical outcome group was 86 years, showing a statistically significant difference according to the *t*-test (*p* = 0.0319) (Table 3). Pearson’s chi-squared test was performed to determine whether the results differed according to gender, but no statistically significant differences were found (χ^2^ (1) = 0.9007, *p* = 0.3426).

In terms of time from onset to arrival, the median time in the favorable clinical outcome group was 52 min, and the median time in the unfavorable clinical outcome group was 57 min, with no statistically significant difference according to the *t*-test and M–W test (*p* = 0.186 and *p* = 0.9214) (Table 3). The median NIHSS score on admission was 20 in the favorable clinical outcome group and 26 in the unfavorable clinical outcome group, showing a statistically significant difference according to both the *t*-test and M–W test (*p* = 0.0005 and *p* = 0.0008) (Table 3). For the ASPECTS and DWI-ASPECTS on admission, in the favorable clinical outcome group, the median ASPECTS was 9, and the median DWI-ASPECTS was 7.5, whereas in the unfavorable clinical outcome group, the median ASPECTS was 9, and the median DWI-ASPECTS was 8, showing no statistically significant differences between the groups according to the *t*-test and M–W test (*p* = 0.0962 and *p* = 0.2867; *p* = 0.3511 and *p* = 0.587) (Table 3). For the NT-proBNP level (pg/mL) on admission, the median for the favorable clinical outcome group was 636.65 (pg/mL), while the median for the unfavorable clinical outcome group was 1195.5 (pg/mL), showing a statistically significant difference according to the M–W test (*p* = 0.0403) (Table 3). For the D-dimer level (µg/mL) on admission, the median for the favorable clinical outcome group was 1.25 (µg/mL), while the median for the unfavorable clinical outcome group was 2 (µg/mL), showing a statistically significant difference according to the M–W test (*p* = 0.0104) (Table 3). Meanwhile, the median HbA1c level (%) on admission was 5.9 (%) in the favorable clinical outcome group and 6 (%) in the unfavorable clinical outcome group, showing no statistically significant difference according to the *t*-test and M–W test (*p* = 0.2431 and *p* = 0.211). There were no statistically significant differences between groups for glucose levels (mg/dl), HbA1c levels (%), or the GAR index at admission in the favorable and unfavorable clinical outcome groups according to *t*-test and M-W tests (*p* = 0.1199, *p* = 0.2431, *p* = 0.2782; *p* = 0. 1347, *p* = 0.2110, *p* = 0.1620). (Table 3).

On further investigation, in the binomial logistic regression analysis, only a lower NIHSS score on admission was significantly correlated with a good clinical outcome (*p* = 0.010).

Although no significant correlations were found, there was a tendency for favorable clinical outcomes to correlate with younger age; NT-proBNP showed a slight tendency to correlate with favorable outcomes (Table 4).

### 3.2. Procedural and Functional Outcomes

Favorable clinical outcomes (mRS ≤ 3) were observed in 47.5% (28/59) of the patients 90 days after admission. The mortality rate within 3 months after the intervention was 20.3% (12/59). There were six deaths from brain herniation due to malignant edema with hemorrhagic infarction, four deaths from aspiration pneumonia, one death from heart failure, and one death from shock due to superior mesenteric artery occlusion.

Successful angiographic recanalization (mTICI ≥ 2b) was achieved in 74.6% (44/59) of the patients. In contrast, unsuccessful recanalization (mTICI 0) occurred in four cases (6.8%). Unsuccessful recanalization cases were due to severe atherosclerotic changes that made it difficult to reach the lesion.

The percentage of successful recanalizations with mTICI 2b or better was 92.9% (26/28) in the favorable clinical outcome group and 58.1% (18/31) in the unfavorable clinical outcome group, showing a statistically significant difference according to both the *t*-test and M–W test (*p* = 0.0018 and *p* = 0.0025) (Table 3).

For the number of attempts, the median for the favorable clinical outcome group was 1 (IQR 1–2), and the median for the unfavorable clinical outcome group was 2 (IQR 1–2), showing a statistically significant difference according to both the *t*-test and M–W test (*p* = 0.0225 and *p* = 0.0244) (Table 3). For the arrival-to-recanalization time (min), the median for the favorable clinical outcome group was 145 (min) (IQR 110.75–178.25), and the median for the unfavorable clinical outcome group was 140 (min) (IQR 117–177), showing no statistically significant difference according to the *t*-test and M–W test (*p* = 0.4178 and *p* = 0.8064) (Table 3). For the puncture-to-recanalization time (min), or the procedure time, the median for the favorable clinical outcome group was 41 (min) (IQR 21.5–57.25), and the median for the unfavorable clinical outcome group was 53 (min) (IQR 36–72), showing no statistically significant difference according to the *t*-test and M–W test (*p* = 0.2049 and *p* = 0.054) (Table 3).

The binomial logistic regression analysis showed no significant correlations, but there was a tendency for cases with TICI ≥ 2b and fewer attempts to correlate with favorable clinical outcomes (Table 4).

## 4. Discussion

In this study, in patients over 80 years of age who underwent MT for LVO, the factors associated with a favorable clinical outcome, according to the *t*-test and M–W test, were younger age, a lower NIHSS score on admission, a lower level of NT-proBNP and D-dimer on admission, the FPE (first pass effect), and successful recanalization. However, the binomial logistic regression analysis showed that only a lower NIHSS score on admission was significantly associated with a favorable clinical outcome. For the other factors, only a trend associated with a favorable clinical outcome was noted.

Many of the younger patients included in various reports of MT outcomes in LVO, including HERMES, had a pre-stroke mRS of 0 [1], and moving from an mRS of 0 to 2 was considered a favorable clinical outcome. For our study group consisting of patients over 80 years of age, the mean pre-stroke mRS was 0.559, which is consistent with moving from an mRS of 0 to 3, and we defined an mRS of 0–3 as a favorable clinical outcome.

No significant gender differences were found in this series, with females having an unfavorable outcome. However, the general consensus seems to be that women have worse functional outcomes than men after ischemic stroke [18].

Similarly, a recent Italian registry of 2399 patients who underwent surgical treatment for chronic limb-threatening ischemia showed that women presented at an older age than males: 79 vs. 73 yo, respectively. Older age, late presentation, delayed diagnosis, smaller-sized vessels, and other sex-related biases have been postulated to account, at least in part, for the portended less favorable outcome in women with some kinds of vascular diseases [19]. Furthermore, one study found that 6 months after stroke, female sex remained an independent predictor of poor prognosis, even after adjusting for other predictors of functional outcomes [20]. Other studies found that 6 months after stroke, female sex remained an independent predictor of poor prognosis, even after adjusting for other predictors of functional outcomes [21]. However, it has also been suggested that if females are more likely to delay treatment for stroke symptoms, this may lead to delays in treatment [22]. However, no clear gender difference was observed in our study.

In particular, hyperglycemia, which is a common phenomenon in acute ischemic stroke patients, has been independently associated with adverse clinical outcomes [23]. Hyperglycemia may promote non-effective recanalization by increasing lactic acidosis, reducing cerebral vasomotor reactivity, and disrupting the blood–brain barrier, resulting in the exacerbation of cytotoxic edema, a reduction in the penumbra, the impairment of the collateral circulation, and an increased risk of symptomatic intracranial hemorrhage (SICH) as consequences [24].

The association between stress hyperglycemia and futile recanalization has already been reported by Merlino et al. [25]. Since GAR is a strong predictor of poor prognosis, they suggest that, in future studies, the management of stress hyperglycemia should not be based on absolute glucose levels but on GAR levels. Furthermore, they suggest paying special attention to patients who have undergone MT for anterior circulation LVO with a GAR index of 17.9 or higher [25]. However, in our study, there were no statistically significant differences in stress hyperglycemia, HbA1c, or the GAR index. The median GAR index was greater than 17.9 for all outcomes. The discrepancy with the point made by Merlino et al. may reflect the fact that the pathophysiology of diabetes is very different between East Asians and Caucasians.

### 4.1. Age

In this study, although there were statistically significant differences in the *t*-test, the binomial logistic regression analysis showed no significant differences; however, it is clear that the older the patient, the more unfavorable the clinical outcome. Similar findings have been reported previously [26,27,28]. As previously noted, pre-existing physical and/or cognitive disability and a high incidence of complications during hospitalization are thought to limit the potential for post-stroke rehabilitation with increasing age [15].

In this study of elderly patients with mRS 6, deaths from infectious complications, such as aspiration pneumonia, were common, while deaths from malignant edema or intraparenchymal hemorrhagic infarction due to stroke were very rare. In the background, many elderly patients have complications such as frailty, and it is thought that their sputum expectoration and other functions are also declining; therefore, it is necessary to pay attention to infectious complications, especially aspiration pneumonia.

In older patients, atherosclerotic changes in the access route are often significant, and treatment may be discontinued because of access difficulties. If successful recanalization is not achieved, a good clinical outcome is unlikely [12].

### 4.2. NIHSS Score on Admission

In this study, the only factor that was found to be significantly associated with favorable clinical outcomes was a lower NIHSS score on admission, and this was found not only in the *t*-test and M–W test but also in the binomial logistic regression analysis. Similar findings have been reported previously [12,29]. Higher NIHSS scores reflect a more extensive cerebral ischemic state and may indicate ischemic symptoms due to the more proximally occluded arteries. Arsava et al. noted the presence of poor collateral circulation [30].

### 4.3. D-Dimer

In this study, there was a statistically significant difference between a high level of D-dimer and an unfavorable clinical outcome in the M–W test but not in the binomial logistic regression analysis. D-dimer is a degradation product of cross-linked fibrin and a biomarker of the fibrinolytic and coagulation systems [31]. Elevated D-dimer levels in acute ischemic stroke have been associated with functional prognosis, acute morbidity, and infarct size [32,33,34,35]. Hisamitsu et al. reported that, in patients with acute stroke, elevated D-dimer levels may be related to increased activation of the fibrinolytic system in the thrombus of the occluded artery, and the larger the thrombus, the greater the increase [36]. Furthermore, they speculated that a high D-dimer level leads to an unfavorable clinical outcome because the amount of thrombus in the occluded artery is high, making it difficult to achieve the first pass effect (FPE), resulting in distal embolization and a prolonged procedure time.

### 4.4. NT-ProBNP

BNP is mainly synthesized by cardiomyocytes in response to increased cardiac chamber wall stress [37]. Activation of BNP produces inactive NT-proBNP (N-terminal pro-brain natriuretic peptide) [38]. NT-proBNP has a longer half-life than BNP and results in less fluctuation, but it has been reported to be associated with adverse clinical outcomes [39,40,41]. Plasma NT-proBNP levels have been reported to be elevated in patients with heart failure, left ventricular dysfunction, acute coronary syndromes, and cardiogenic stroke [42,43]. In addition, NT-proBNP is a biological marker of cerebrovascular disease for identifying ischemic stroke subtypes and predicting the incidence of atrial fibrillation-related stroke [44,45]. In this study, almost all patients with elevated plasma NT-proBNP had a cardiogenic embolism. Furthermore, there is increasing evidence that serum levels of NT-proBNP are associated with an increasing risk of adverse clinical outcomes in patients after stroke [40,46].

There are no reports on the association between NT-proBNP and prognosis in MT for LVO in elderly patients. Chronic heart failure has been reported to exacerbate cerebrovascular disease, dementia, and pneumonia and to reduce cardiac activity [47,48,49].

In this study, although there were statistically significant differences in the association of the FPE with favorable clinical outcomes in both the *t*-test and the M–W test, there were no significant differences in the binomial logistic regression analysis. The results suggest that cardiac function also contributes to improved prognosis after rehabilitation. Therefore, cardiac function may be an indicator of functional prognosis after MT for LVO.

In this study, NT-proBNP levels were elevated well above 150 ng/mL, which is considered a marker for heart failure. However, most patients had no obvious symptoms of heart failure. In a case–cohort study of the REGARDS cohort, the authors confirmed that the association of NT-proBNP with stroke was stronger for cardioembolic stroke [50].

Zhang et al. proposed three possible mechanisms of NT-proBNP elevation: (1) Central blood vessel damage promotes the massive release of NT-proBNP from the central nervous system (CNS) to the blood, resulting in an absolute increase in NT-proBNP. (2) NT-proBNP concentrations derived from the same precursor protein increase simultaneously when cerebral infarction occurs; however, because of their different patterns of metabolism, the activation of the endocrine system controlled by the CNS degrades BNP more than NT-proBNP, resulting in a relative increase in NT-proBNP. (3) Following the deterioration that occurs because of heart failure, organisms begin to mobilize activity factors that collaborate with the renin–angiotensin–aldosterone system to induce a diastolic state in the blood vessels [51].

In this study, the elevation in NT-proBNP levels in LVO not only aligned with one of the above three possibilities but was also the result of a sudden increase in blood pressure that promoted the spontaneous recanalization of the large vessel occlusion, which may have resulted in an excessive cardiac load.

The cases with elevated levels of NT-proBNP and unfavorable clinical outcomes included those who died because of complications such as heart failure or aspiration pneumonia during the course of hospitalization. Some cases were already complicated by aspiration pneumonia at the onset of hospitalization, which may have played a role in the elevated NT-proBNP levels.

Many of the cases with elevated levels of NT-proBNP were consequently older and with a higher NIHSS, and the elevation may have been the result of greater sympathetic activation.

In the present study, the regression analysis failed to produce significant results because of the insufficient power of the sample size. Therefore, if the sample size is increased, the regression model may be able to produce results showing that NT-proBNP is an independent predictor of prognosis.

### 4.5. FPE

In this study, although there were statistically significant differences in both the *t*-test and the M–W test, the binomial logistic regression analysis showed no significant differences. Zaidat et al. showed that patients with mTICI 3 had better clinical outcomes, lower mortality, and fewer procedural adverse events after one pass [52]. Rousiers, E.D., et al. reported that the FPE is a significant determinant of good clinical outcomes in the oldest patients. Similar effects were observed in our results [10]. The FPE has been shown to reduce the procedure time, ischemic core volume expansion, and the incidence of procedural complications [53,54]. However, our results on the puncture-to-recanalization time showed no correlation with clinical outcomes.

## 5. Limitations

Nevertheless, the present study has some limitations. The study design was retrospective and non-randomized with a small sample of patients treated at a single institution. We did not compare patients treated with MT with those treated with medical treatment including thrombolysis alone. We did not perform serial measurements of NT-proBNP and D-dimer levels; therefore, we were unable to examine the association of changes in these levels during the phase after acute stroke. In the future, patient data should be accumulated, and a large-scale prospective study should be conducted. Although the regression analysis did not produce significant results because of the insufficient power of the sample size, a certain trend was observed; therefore, if the sample size is increased in the future, it may be possible to produce results for an independent prognostic predictor using a regression model.

## Figures and Tables

**Table 1:** Patients’ characteristics.

	Favorable Clinical Outcome 28 Cases	Unfavorable Clinical Outcome 31 Cases
Hypertension	14 (50%)	25 (80.65%)
Diabetes	4 (14.29%)	8 (25.81%)
Hyperlipidemia	5 (17.86%)	3 (9.68%)
Atrial fibrillation	24 (85.71%)	19 (61.29%)

*t*-test and M–W test (*p* = 0.7802 and *p* = 0.7728).

**Table 2:** Occlusion site.

	Favorable Clinical Outcome 28 Cases	Unfavorable Clinical Outcome 31 Cases
Cervical ICA	0	4
Intracranial ICA	1	2
Terminal ICA	6	7
M1 proximal	6	3
M1 distal	8	10
M2/M3	7	4
Tandem	0	1

ICA: internal carotid artery; M1: middle cerebral artery horizontal segment; M2/M3: middle cerebral artery insular segment/opercular segment; tandem: sub-occlusion or stenosis of extracranial internal carotid artery, together with simultaneous intracranial large vessel occlusion. *t*-test and M–W test (*p* = 0.8129 and *p* = 0.7481).

**Table 3:** Prognosis factor analysis.

	Favorable Clinical Outcome 28 Cases	Unfavorable Clinical Outcome 31 Cases	t-Stats	*p*	M–W Test	*p*
Age, median (IQR) years	84 (82.75–86)	85 (83–90)	−2.1998	0.03189 *	329	0.1107 *
Female	11 (39.29%)	16 (51.6%)				
CT ASPECTS, median (IQR)	9 (7–9.25)	9 (7–9)	−3.6873	0.0962	502.5	0.2867
MRI DWI-ASPECTS, median (IQR)	7.5 (6–9)	8 (6–8)	1.9157	0.3511	381.5	0.587
NIHSS, median (IQR)	20 (16–24.25)	26 (22.5–30)	−3.6873	0.00051 *	213 *	0.0008 *
Intravenous thrombolysis	18 (64.29%)	18 (58.06%)				
Glucose, median (IQR) (mg/dL)	114 (101–146)	131 (109–162)	−1.5799	0.1199	−7.74	0.1326
HbA1c median (IQR) (%))	5.9 (2.2–6)	6 (5.625–6.475)	−1.5017	0.1389	−27.41	0.3481
GAR index, median (IQR)	19 (18–23)	23 (19–26)	−1.0872	0.2817	−34.64	0.1463
D-dimer median (IQR) (µg/mL)	1.25 (0.875–1.8)	2 (1.2–4.05)	−0.70065	1.4864	255	0.01037 *
NT-proBNP median (IQR) (pg/mL)	636.65 (314.2–913)	1195.5 (746.6–1814)	−1.6016	0.1164	169	0.04028 *
TICI2b <	26 (92.86%)	18 (58.06%)	3.28524	0.00175 *	585	0.002457 *
Number of passes, median (IQR)	1 (1–2)	2 (1–2)	2.34518	0.02252 *	562	0.024427 *
Groin-to-recanalization time, median (IQR) (min)	41 (21.5–57,25)	53 (36–72)	−1.28237	0.20491	307	0.05475
Arrival-to-recanalization time, median (IQR) (min)	145 (110.75–178.25)	148 (117–177)	0.816	0.4178	−0.251	0.8064
Onset-to-arrival time, median (IQR) (min)	52 (38.75–165.75)	57 (39.5–157)	1.3385	0.186023	441	0.9214

CT ASPECTS: Alberta Stroke Program Early CT Score; MRI DWI-ASPECTS: Diffusion-Weighted Imaging ASPECTS; NIHSS: National Institutes of Health Stroke Scale; HbA1c: glycated hemoglobin; GAR index: glucose-to-glycated hemoglobin ratio; NT-proBNP: N-terminal pro-brain natriuretic peptide; mTICI: modified thrombolysis in cerebral infarction; *t*-test: Student’s *t*-test; M–W test: Mann–Whitney U test; *: statistically significant *p* < 0.05.

**Table 4:** Binomial logistic regression model analysis of prognostic factors.

	B	Standard Error	Wald	*p*	Exp(B)
Age	−0.164	0.125	1.712	0.191	0.849
NIHSS score on admission	−0.183	0.076	5.749	0.017 *	0.833
NT-proBNP	−0.001	0.000	1.678	0.195	0.999
D-dimer	−0.046	0.99	0.214	0.644	1.047
Number of passes	−0.541	0.578	0.876	0.349	0.582
mTICI ≥ 2b	2.694	1.377	3.825	0.050	14.788
Constant	16.626	11.065	2.258	0.133	16,609,785.569

NIHSS: National Institutes of Health Stroke Scale; NT-proBNP: N-terminal pro-brain natriuretic peptide; mTICI: modified thrombolysis in cerebral infarction; *: statistically significant.

## Data Availability

The data that supporting the results of this study are not publicly available because they contain information that could compromise the privacy of the research participants. All data are presented in this manuscript. Data are not stored in repositories or any other source.
